# Validating the fever protocol – Acute undifferentiated febrile illness in the ED

**DOI:** 10.1186/1865-1380-8-S1-P8

**Published:** 2015-04-22

**Authors:** Sivaraj Durairaj, Parivalavan Rajavelu, Tausif Thangalvadi

**Affiliations:** 1 Sundaram Medical Foundation, Chennai, India

## Objective

Fever is the most common complaint presenting to our ED affecting 1 in 5 patients. In the absence of literature for acute patients presenting to the ED with fever, we had done a pilot study in 2010, using a protocol based approach to manage undifferentiated febrile illness in the ED which advocates judicious use of investigations and antibiotics. There is a need to prospectively validate the appropriateness of this fever protocol. The purpose of this study is to validate the fever protocol for the evaluation and treatment of acute undifferentiated febrile patient who have no localizing symptoms in order to reduce unnecessary testing and inappropriate antibiotic use.

## Methods

The study was done at Sundaram Medical Foundation, a 200 bedded community hospital in Chennai. This was a prospective study done from May 2014 to July 2014. Inclusion criteria were all adult patients age >18 presenting to the ED with fever. Exclusion criteria were patients with fever with localizing signs, sepsis, investigations done and antibiotics given outside SMF. As per the fever protocol, investigations were based on duration of fever with no investigations prior to day 3. Treatment was guided by results of investigations. Patients were asked to review on 3^rd^ day if fever persisted and reviewed over phone on 30^th^ day. Data was collected using structured data sheet. Data was analyzed using Excel and presented as proportions.

## Result

A total of 421 patients were recruited in the study out of which 159 were eligible for inclusion. N=159, Male:Female ratio 46:54. The most common age group was 18-40 yrs (68.5%). Majority of the patients presented on Day 1 or 2 of fever (n = 94, 59%), 49 (31%) presented on Day 3 or 4 of fever and 16 (10%) presented on or after Day 5. Sixteen patients were lost to follow-up. Based on the fever protocol, 65 patients (41%) needed investigations on first visit and 5 (3%) needed admission. Out of those followed up, 77 (54%) did not need a revisit to the ED. Out of the 94 patients who presented with fever<3days, only 7 needed investigations on a revisit. Out of the 66 who revisited the ED, 42% (28) needed further investigations and 15% (10) got admitted. Only 22 patients (14%) needed antibiotics during the course of treatment. All patients followed up had recovered on 30-day follow-up. More than half (59%) recovered within 4 days of fever, 30% needed 7 days for recovery and only 11% took more than 1 week. Most common suspected cause of fever was Viral. Enteric fever was the most common reason for starting antibiotic with Cefixime being the most common antibiotic used.

## Conclusion

Majority of patients (86%) presenting to the ED with undifferentiated febrile illness do not require antibiotics and only 1 out of 10 needed admission. Nine out of ten patients presenting to the ED on Day 1 or 2 of fever defervesced without the need for further investigations. The fever protocol enables the judicious use of investigations and antibiotics decreasing cost and resistance to antibiotics.

